# Fast cars, slow words? Tracking processing of concurrent speaking and driving in real time

**DOI:** 10.3758/s13423-026-02954-z

**Published:** 2026-09-03

**Authors:** Marie Hansen, Pamela Fuhrmeister, Agnieszka Hearder, Audrey Bürki

**Affiliations:** 1 Department of Linguistics, University of Potsdam, Karl-Liebknecht-Str. 24-25, 14476 Potsdam, Germany; 2 Institute of Psychology, University of Lausanne, Géopolis, 1015 Lausanne, Switzerland

**Keywords:** Dual-task performance, Cognitive and attentional control, Language production, Psycholinguistics

## Abstract

The study of multitasking provides a unique window into the interaction of cognitive processes and systems and as such, into the cognitive architecture of the human mind. Previous studies reported that when participants speak and do something else at the same time, task performance often suffers. In the present study, we examine the mechanisms leading to such interference in multitasking in an ecological setting. We compare speaking performance across easy and difficult driving tasks and track measures of speech fluency and driving efficiency throughout the experiment. Interference is observed only under difficult driving conditions. Moreover, fluctuations in speaking performance predict fluctuations in driving performance and vice versa. When speaking efficiency decreases, so does driving efficiency. These findings have a straightforward explanation in accounts of dual-task interference, in which interference arises when task demands exceed available cognitive resources. They further suggest that when this limit is reached, the system downregulates resources on both tasks.

## Introduction

Speaking mostly feels effortless. Yet, when we do something else at the same time, speech fluency often suffers. Empirical studies corroborate this observation. In these studies, participants produce connected speech (e.g., in response to prompts) in isolation and while performing an unrelated task. Speech fluency in one condition is compared to fluency in the other. Speech fluency decreases when participants speak while performing another task, whether the concurrent task is artificial (e.g., speaking with concurrent motion tracking or tactile form recognition, Kemper et al., 2009; Oomen & Postma, 2001) or more ecological. For instance, Glenn (2017) and Dromey and Simmons (2019) report that speech fluency is lower when participants respond to prompts during simulated driving than without a concurrent task. Moreover, in studies in which participants speak and drive, driving tends to be less efficient during concurrent speaking (Dromey & Simmons, 2019; Glenn, 2017; Horrey & Wickens, 2006; Strayer & Johnston, 2001). These findings suggest that language planning relies on cognitive resources or processes that are also required to perform non-linguistic tasks but they do not inform on why interference occurs, i.e., on the cognitive processes and mechanisms underlying the performance decline. The aim of the present study is to address this issue. We extend previous studies by manipulating task difficulty^1^ and by moving beyond comparisons of mean performances across conditions, to examine the temporal coupling of on-going speaking and driving performance over the course of the experiment.

The mechanisms underlying interference while speaking have been studied in highly controlled experiments, such as the Psychological Refractory Period Paradigm (PRP, Smith, 1967; Welford, 1952). In this paradigm, two types of stimuli are presented in rapid succession on each trial, and participants are requested to perform a different task with each of them while prioritizing Task 1. The delay between the presentation of the two stimuli is varied. Slower responses to Task 2 at short delays indicate interference (Ferreira & Pashler, 2002; Piai & Roelofs, 2013; Roelofs, 2008). Building on this work, we ask whether the mechanisms proposed to explain interference in PRP tasks (see below) can also account for the interference observed in more ecological settings involving continuous, rather than trial-by-trial, tasks. Settings in which participants produce connected speech while continuously performing a concurrent task (e.g., driving) differ from artificial trial-by-trial settings in important respects. Participants are not instructed which task to prioritize, and events unfold less predictably. Moreover, the type of language task differs. In trial-by-trial experiments, participants produce small speech units (usually single words) in response to discrete stimuli. This is necessary to enable tight control over the timing and content of responses, such that the time course of planning processes can be inferred from response times. By contrast, continuous settings allow the use of language tasks that elicit connected speech, making them more similar to everyday language use. It has been suggested that interference depends on different factors, including the type of task (Meyer & Kieras, 1997; Norman & Shallice, 1986), task instructions (Navon & Miller, 2002), and task characteristics such as the order of the presented stimuli (Miller et al., 2009; Schumacher et al., 1999). Consequently, the mechanisms leading to interference may differ between continuous and trial-by-trial settings.

As mentioned above, several theoretical accounts have been put forward to explain dual-task interference in trial-by-trial paradigms, and the factors that influence it (Hazeltine et al. 2006; Pashler 1994; Ruthruff et al. 2003). In the structural bottleneck account, some steps required for performing each task recruit the same processes, which can only be used by one task at a time (serial processing) due to structural constraints in the system (Pashler, 1994; Welford, 1967). According to alternative views, serial processing may occur (depending on task characteristics, task combinations, or instructions) but is not structural (and therefore not mandatory). Accounts in which there is no structural bottleneck propose different mechanisms to explain interference. Some assume that interference arises because participants opt for serial processing in some settings, for instance, to avoid costly conflicts between concurrent processes (Meyer & Kieras, 1997; Roelofs, 2008) or to minimize task completion times when task stimuli are presented sequentially (Miller et al., 2009). Others propose that interference is not a consequence of serial processing. Processes operate in parallel, and interference occurs when the resources needed to perform the two tasks efficiently no longer suffice (Mittelstädt et al., 2022; Tombu & Jolicoeur, 2002, 2003). Finally, some authors suggested that interference stems from costs related to the monitoring of task performance and the managing of limited resources (supervisory attentional system, see Norman and Shallice 1986; see also Mittelstädt and Miller 2017, as well as Pashler 1994, for a related discussion of preparational demands in multitasking compared to single tasks).

The data available to date suggest that different mechanisms have a psychological reality (for evidence in favor of either seriality or limited resources, see, e.g., De Jong 1993; Pannebakker et al. 2011; Ruthruff et al. 2001; Schumacher et al. 1999; Tombu and Jolicoeur 2003. It has further been suggested that a structural bottleneck only occurs in specific situations (e.g., two tasks both entail the selection of a response, McCann & Johnston, 1992; Pashler, 1994) and that the PRP paradigm may bias participants toward serial processing (Miller et al., 2009; Navon & Miller, 2002).

The aim of the present study is not to resolve the theoretical debate around serial versus parallel processing that took place in the PRP literature, or to find a universal explanation for interference, but to shed light on the mechanisms leading to interference when speakers produce connected speech and perform another task at the same time in an ecological setting. Ultimately, the aim of cognitive science should be to understand how the cognitive system functions in everyday settings.

Our participants responded to prompts (e.g., *Describe your typical Sunday.*) while driving in a simulator. We tracked speech fluency with measures that have been shown to index language planning (Barr, 2001; Bortfeld et al., 2001; Butterworth, 1975; Goldman-Eisler, 1968; Peters et al., 2024; Tavakoli et al., 2020) and to be impacted by a concurrent task (Dromey & Simmons, 2019; Glenn, 2017; Jou & Harris, 1992; Kemper et al., 2009). We further recorded variability in velocity and lateral position of the car on the road, measures that have been related to cognitive control and attention during driving (Dromey & Simmons, 2019; Kubose et al., 2006; Rakauskas et al., 2004). We monitored speaking performance in speaking-only, in an easy driving scenario, and in a difficult driving scenario. In the driving scenarios, we tracked the temporal coupling of speaking and driving over the course of the experiment.

In the light of the above, the following scenarios can be considered to explain interference from a concurrent task during speaking in our setting. In a first scenario, a structural bottleneck delays some planning processes (e.g., lemma selection, see Ferreira and Pashler, 2002), resulting in decreased fluency. When producing connected speech, speakers constantly select responses (i.e., words) and an appropriate timing of word selection ensures fluent speech. If the driving task requires processes that are also needed for speaking (e.g., selecting responses), word selection may be delayed, leading to disfluencies. Under this scenario, we expect a performance decline in speaking while driving compared to isolated speaking and possibly even more interference when driving is difficult (assuming that the number of responses to select increases). Moreover, driving and speaking performances should correlate negatively. When a process is occupied by one of the two tasks, processing in that task is efficient, whereas processing in the other task deteriorates.

In a second scenario, some processes required by the two tasks cannot operate efficiently in parallel, but the bottleneck can be avoided in continuous settings through flexible task scheduling. Therefore, participants opt for serial processing to minimize conflicts between concurrent processes (Roelofs, 2008). While task scheduling may be sufficiently efficient to prevent interference when the concurrent task is easy, efficiency may decrease when the number of potentially overlapping processes increases, leading to interference. Under this scenario, interference is expected in the more demanding but not necessarily in the easy driving condition. A negative correlation is expected under difficult driving conditions.

In a third scenario, interference is not generated by serial processing (irrespective of whether serial processing does, may, or must occur in this or other settings) but by the fact that sometimes, multitasking demands more resources than are available. In one variant of this scenario, available resources are solely used to perform the tasks in parallel (Mittelstädt et al., 2022; Tombu & Jolicoeur, 2002, 2003). In another variant, at least part of the available resources is required to manage or supervise task performance (Norman & Shallice, 1986). Either way, more interference is expected in difficult driving, as it requires more resources. Whether task performance covaries depends on the adopted strategy when reaching maximum capacity. The system could reduce resources for both tasks, leading to simultaneous performance declines and a positive correlation. Alternatively, the system could sometimes prioritize one task and sometimes the other. In this case, performance on the two tasks is not expected to covary. A strategy in which participants prioritize one of the two tasks throughout the experiment is unlikely, given previous evidence of reciprocal interference between speaking and driving.

## Methods

### Participants

Twenty-five German native speakers without cognitive or neurological disorders and in possession of a German driver’s license participated in the experiment. The sample size corresponds to the number of participants that we could afford to test with our resources. Data from five participants had to be excluded due to technical failures in the audio recordings or in the driving simulator sampling. The data of 20 participants were analyzed (14 females, six males, mean age = 24.10, SD = 3.26). This sample size is similar to that used in similar studies (Dromey & Simmons, 2019; Glenn, 2017).

### Procedure

We used the Carnetsoft driving simulator (Groningen, NL) located inside a soundproof booth. Participants could hear the experimenter’s voice inside the booth through a speaker. First, participants were familiarized with the driving simulator. They then performed a practice drive and a driving automaticity test (Peripheral Detection Task; PDT, Martens & Van Winsum, 2000). Three experimental conditions followed: speaking during an easy driving task, speaking during a hard driving task, and speaking-only (without driving). Easy driving was always completed before hard driving but the position of the speaking-only task was rotated across participants. We decided on this design based on findings from previous studies in our lab, which showed that the difficulty of a task impacts performance in a subsequent task. Consequently, however, participants had more practice in the difficult driving task, because they had already performed the easy driving scenario. The speaking task was the same across conditions: The experimenter prompted participants to speak freely about a given subject (i.e., routine, familiar place, experience, preference). There were three prompt lists (see Appendix A) rotated across conditions. In the driving conditions, prompts were given after reaching a speed of 80 km/h. Each task ended after recording seven minutes of speech. Easy driving consisted of a driving scenario without traffic or intersections. Difficult driving required driving on the left side of the road (Germany is a country with right-hand traffic), with oncoming traffic and road signs (that did not require participants to brake).

We excluded portions of the audio recordings corresponding to comments on the task, problems with the driving task, or where the experimenter was speaking. Word boundaries were annotated with the Montreal Forced Aligner (McAuliffe et al., 2017) and hand-corrected by trained researchers using Praat (Boersma & Weenink, 2024).

### Analyses

We did not preregister this study but decided on the analysis plan and the dependent variables before looking at the data. Analyses that were added after deciding on the original analysis plan are referred to as *exploratory analyses*. Data and scripts are available on the Open Science Framework (see Open practices statement).

Following previous driving simulator studies (Dromey & Simmons, 2019; Glenn, 2017), we selected two measures of speech fluency: speech ratio and mean utterance length. The first reflects the frequency and length of disruptions such as silent pauses and hesitations, while the latter captures aspects of syntactic complexity (e.g., Kemper et al., 2003; Terken & Collier, 1992). Speech ratio was calculated as the ratio of the time spent speaking to the total time of the speaking task. To obtain a measure of mean utterance length, we counted the number of morphemes within any speech between silent pauses using the Python package *spaCy* (Honnibal & Montani, 2017). Two additional measures, speaking rate and the number of filled pauses, were considered in exploratory analyses. Speaking rate was defined as the number of syllables per second (De Jong & Wempe, 2009; Kim & Nam, 2008; Wang & Narayanan, 2007). Filled pauses consisted of *äh* (uh), *ähm* (uhm), *hmm*, and *mmm* (Oomen & Postma, 2001; Schachter et al., 1991; Wingate, 1984) and were identified using spaCy’s Matcher function. To obtain measures of driving efficiency, we further followed Dromey and Simmons (2019) as well as Glenn (2017) and computed the variability (standard deviation) of velocity and lateral position of the car on the road. A lower variability in these measures has been associated with better driving performance (e.g., Dromey & Simmons, 2019; Glenn, 2017; Kubose et al., 2006; Rakauskas et al., 2004).

A first series of analyses aimed at assessing the impact of condition on language planning. We ran mixed-effects regression models using speech fluency measures as dependent variables. We used linear models to analyze speech ratio and Poisson regression to analyze mean utterance length, speaking rate, and the rate of filled pauses relative to the time spent speaking. In all models, contrasts were set to test the difference between the two driving conditions as well as the difference between speaking-only and the mean of the driving conditions (Helmert coding). The average response time in the PDT task was added as a control predictor. The models included by-participant random intercepts and were run using the R package *lme4* (Bates et al., 2015). We also ran exploratory models with treatment contrasts comparing the dependent variables in the speaking-only condition with those in each driving condition.

A second series of analyses aimed at assessing the co-variation of speaking and driving efficiency over time. We split the data into time windows of 5 s and computed speech ratio and variability in driving measures in each time window. First, we used panel Granger causality analysis (Dumitrescu & Hurlin, 2012; Granger, 1969; Lopez & Weber, 2017) to test whether driving measures improved the prediction of speech ratio in subsequent time windows beyond its own past values and vice versa, while accounting for repeated measures across participants. Second, we used linear mixed models to predict speech ratio as a function of driving condition, variability in driving measures, and their interaction^2^. We also included a predictor *time*, reflecting the order of the time bins used for the computation of variability measures (i.e., time bin 1 to n) as well as its interactions with driving performance measures. This was done to control for potential effects of practice or fatigue. Driving measures were centered on the mean, and driving condition was coded as a scaled-sum contrast, so the estimate represents the difference between the two conditions. The models were run with correlated by-participant varying intercepts and slopes for the effect of condition.

**Table 1:** Differences in speech ratio between conditions (Helmert coding)

*Predictors*	*Estimate*	*SE*	*p*
Intercept	1.38	0.05	<.001
Speaking only - Driving	0.03	0.04	0.473
Easy driving - Hard driving	0.16	0.04	<.001
Response time PDT	0.08	0.81	0.922

**Table 2:** Differences in speech ratio between conditions (treatment coding)

*Predictors*	*Estimate*	*SE*	*p*
Intercept	1.40	0.06	<.001
Easy driving - Speaking only	0.06	0.04	0.183
Hard driving - Speaking only	-0.11	0.04	0.009
Response time PDT	0.08	0.81	0.922

## Results

### Speech fluency as a function of condition

**Fig. 1:**
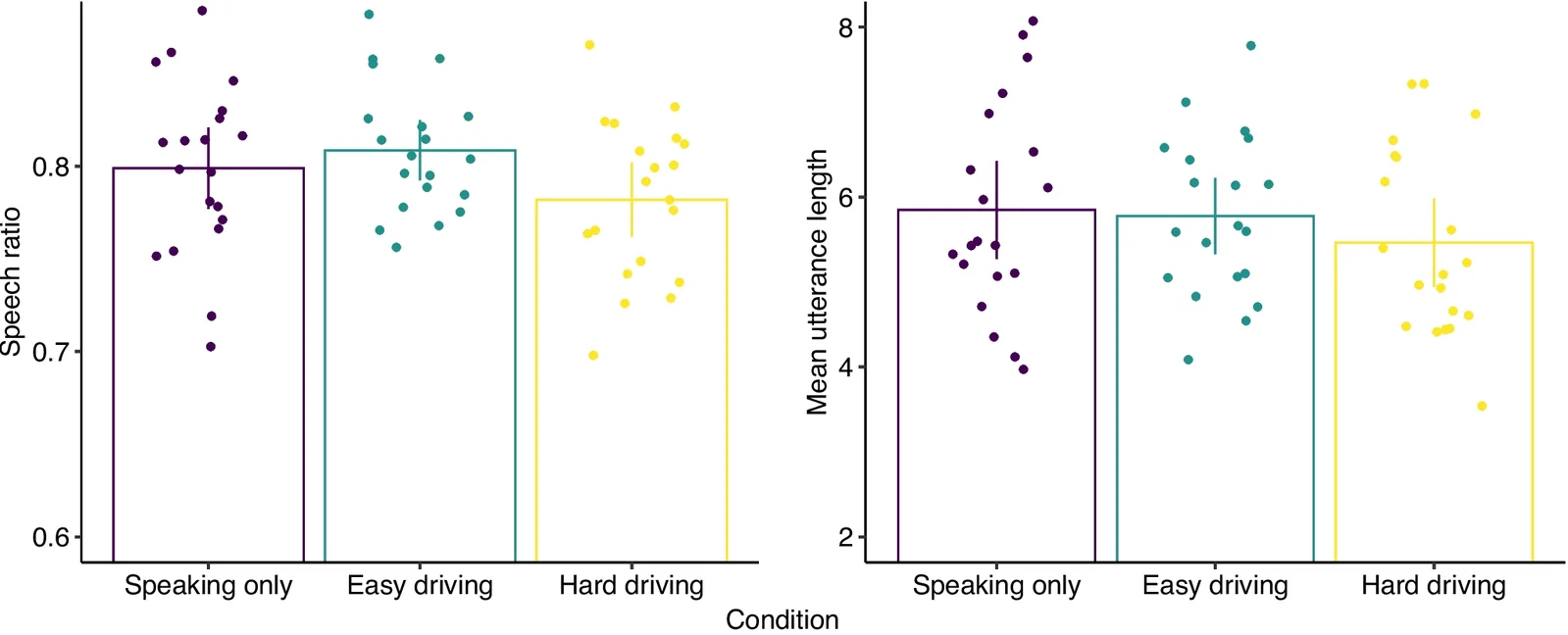
Plots showing speech ratio and mean utterance length per condition. Individual points represent individual participants. *Error bars* represent 95% confidence intervals

Speech ratio was 0.80, 0.81, and 0.78 in the speaking-only, easy driving, and hard driving conditions, respectively. Speech ratio was significantly higher in the easy condition than in the hard condition, indicating that participants made more or longer silent pauses in the hard condition. There was no significant difference between the speaking-only and the mean of the two driving conditions (Table 1). The model comparing the speaking-only to each of the driving conditions only showed a difference between speaking-only and hard driving (Table 2, Fig. 1). Effect sizes are in the range of similar studies (0.07 and 0.03, respectively, in Dromey & Simmons, 2019; Glenn, 2017).

Mean utterance length was 5.85, 5.78, and 5.46 in the speaking-only, easy driving, and hard driving conditions, respectively. Utterances were significantly longer in speaking-only compared to the mean of the two driving conditions and in easy compared to hard driving (Table 3, Fig. 1). The model comparing the speaking-only to each of the driving conditions revealed longer utterances in speaking-only compared to hard driving (Table 4) but no difference between speaking-only and easy driving.

Results of exploratory analyses of speaking rate and number of filled pauses are reported in Appendix B. There was no significant difference between conditions for these measures.

### Relationships between driving and speaking measures

**Fig. 2:**
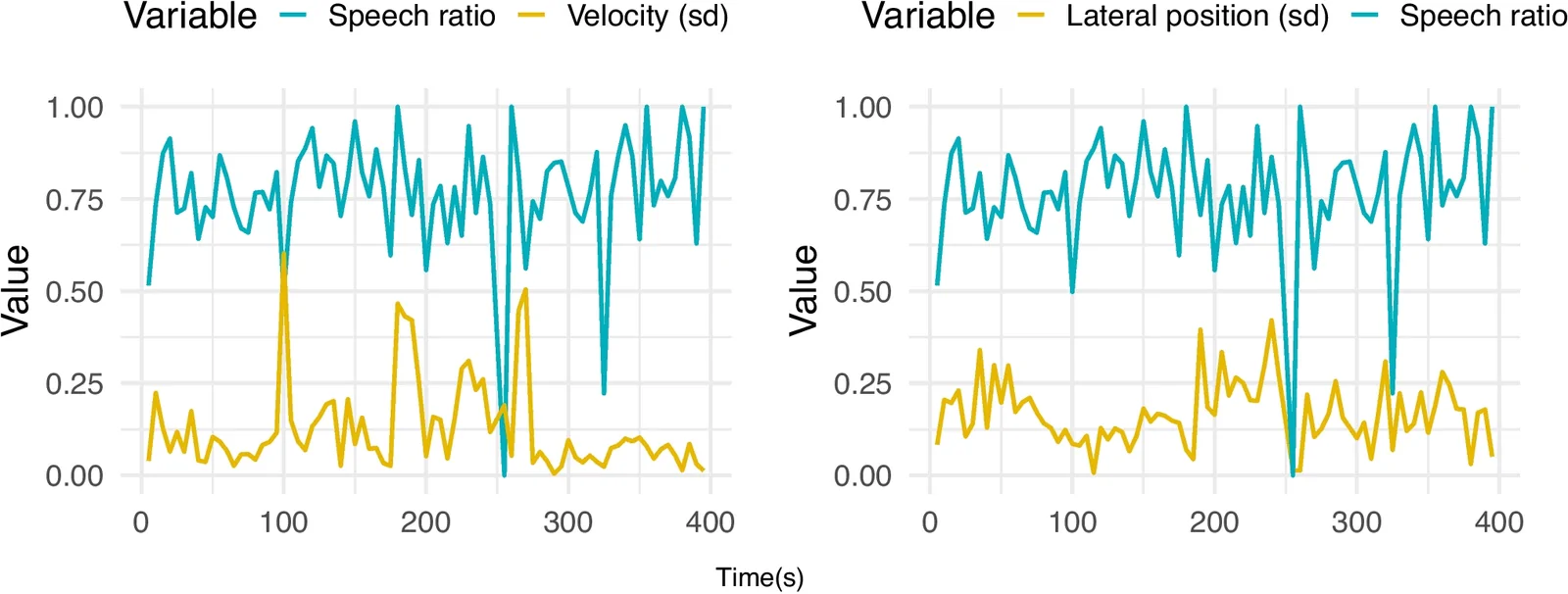
Plots showing the covariation of the time series of speech ratio and variability in velocity (*left side*)/ variability in lateral position (*right side*), averaged over 5-s intervals for one participant in the hard driving condition

**Fig. 3:**
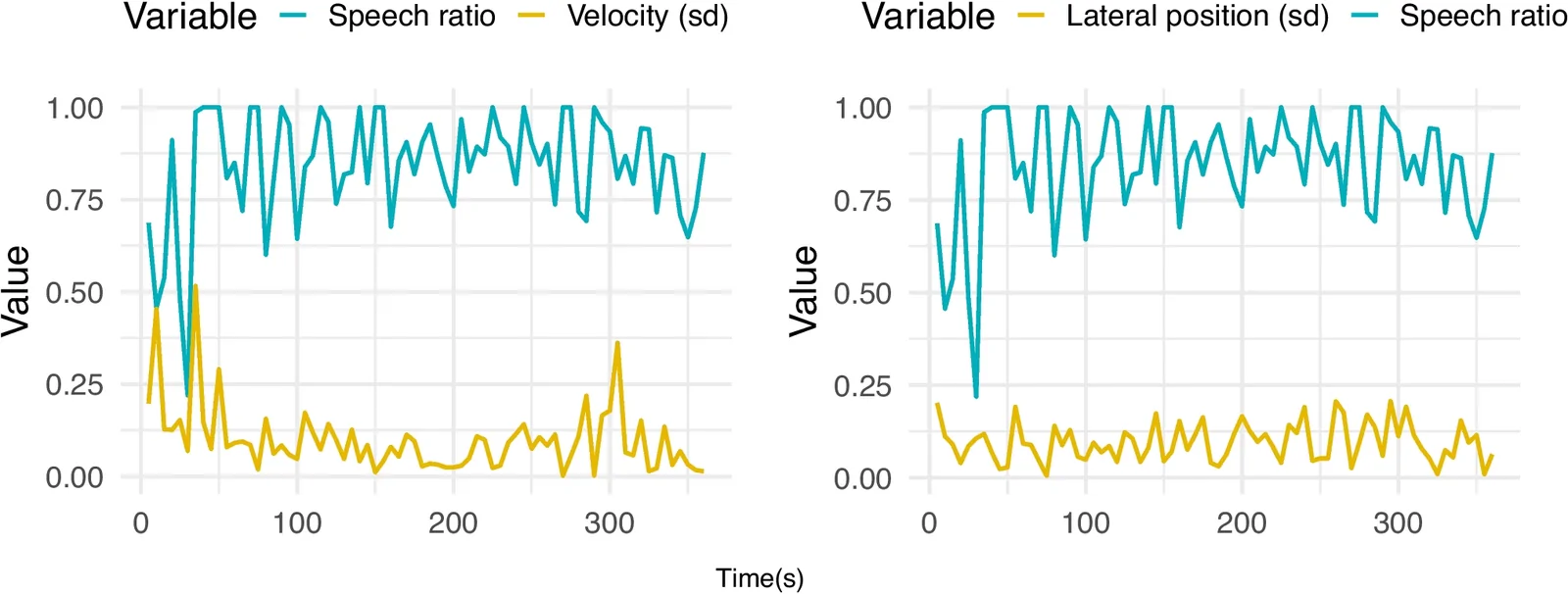
Plots showing the covariation of the time series of speech ratio and variability in velocity (left side)/ variability in lateral position (*right side*), averaged over 5-s intervals for the same participant in the easy driving condition

**Fig. 4:**
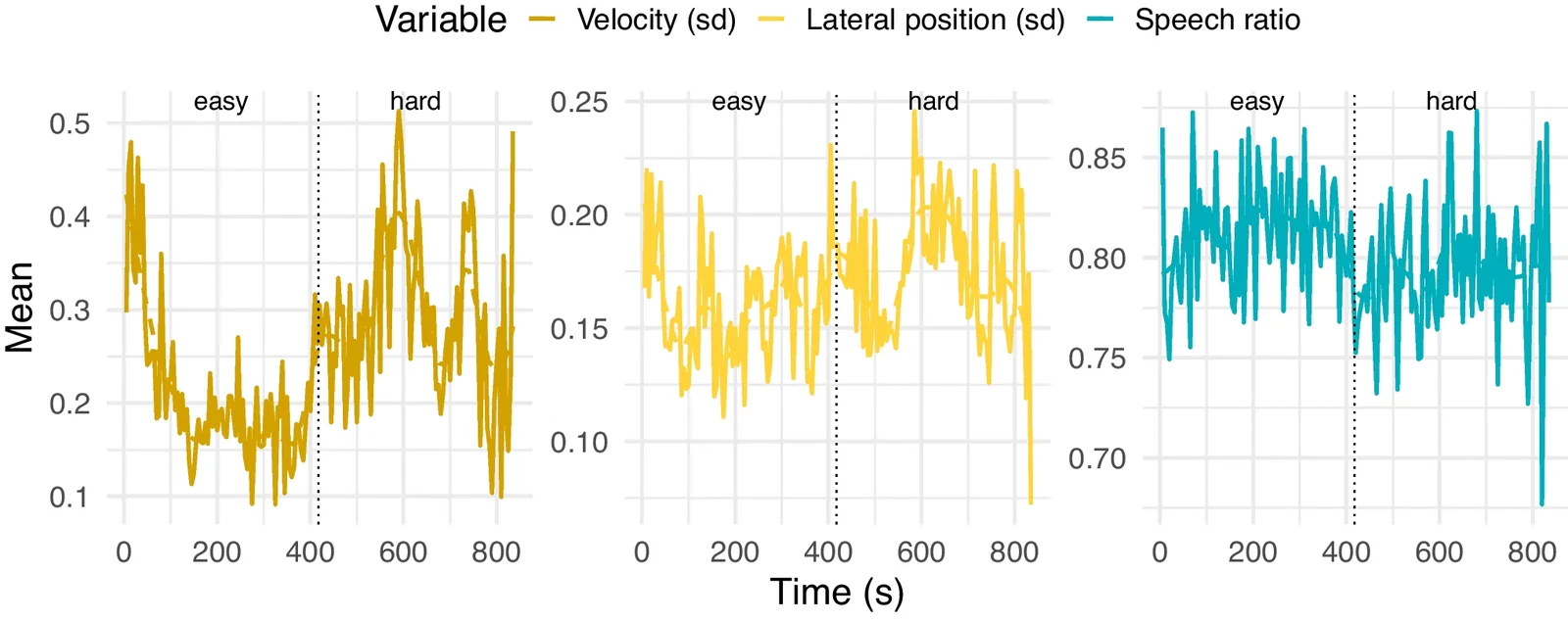
Plots showing time series of speech ratio, variability in velocity, and variability in lateral position, averaged over 5-s intervals, across participants. The easy driving task was performed in the first half of the experiment, the hard driving task in the second half

Figures 2, 3, and 4 illustrate the variation of speech ratio and variability in driving measures over time, for individual participants in the hard (Fig. 2) and easy condition (Fig. 3), and across participants and conditions (Fig. 4). Granger causality analyses showed that variability in velocity and lateral position *Granger caused* speech ratio and vice versa, but only in the hard driving condition (Table 5).

**Table 3:** Differences in mean length of utterance between conditions (Helmert coding)

*Predictors*	*Estimate*	*SE*	*p*
Intercept	1.72	0.04	<.001
Speaking only - Driving	0.05	8.77e-03	<.001
Easy driving - Hard driving	0.06	0.01	<.001
Response time PDT	0.07	0.57	0.9

Linear models predicting speech ratio in 5 s time bins revealed that variability in lateral position had a negative impact on speech ratio. In addition, as in the first analysis, speech ratio was significantly higher in easy compared to hard driving. There was no significant effect of time. Neither driving condition nor time interacted with variability in lateral position or velocity^3^ (see Table 6).

## Discussion

Explaining the cognitive architecture of the human mind requires understanding how the different cognitive processes and subsystems relate to one another. The use of multitasking studies for this purpose has a long tradition in cognitive psychology (dating back to Smith, 1967; Telford, 1931; or Welford, 1952). The present study focused on an everyday multitasking situation, where participants freely coordinated the production of connected speech with continuous driving. We aimed to explain the interference observed in this setting, building on mechanisms proposed to explain interference in highly constrained dual-task experiments. Participants were requested to speak and drive in a simulator. Speech ratio and mean utterance length decreased under difficult driving conditions compared to easy driving and isolated speaking.^4^ Analyses further revealed a positive relationship between task performances – when speaking performance declined, driving efficiency also decreased.

**Table 4:** Differences in mean length of utterance between conditions (treatment coding)

*Predictors*	*Estimate*	*SE*	*p*
Intercept	1.75	0.04	<.001
Easy driving - Speaking only	-0.02	0.01	0.139
Hard driving - Speaking only	-0.08	0.01	<.001
Response time PDT	0.07	0.57	0.9

These findings constrain the range of possible explanations for the observed interference of driving on speaking. They are consistent with a view under which interference occurs when the maximum capacity of available resources is reached. The more difficult condition is expected to require more resources and therefore, maximum capacity is reached more often. While capacity-limited accounts unambiguously assume that interference arises whenever the available resources are exceeded, it is less clear how the cognitive system behaves at this limit. The system could either alternate resource allocation between tasks or decrease resources for both tasks. The present findings provide some insight into this question. The positive relationship between performance measures suggests that in our setting, the system reduces the resources on both tasks, causing simultaneous performance declines in both.

Our findings are more difficult to reconcile with a scenario where interference is due to a structural bottleneck. The observation that interference emerged only in difficult driving could be accounted for by assuming that such a bottleneck can be avoided by flexible task scheduling in the easy but not the difficult condition (Roelofs, 2008), or by assuming that the bottleneck only arises in the latter. As outlined in the Introduction, some authors have argued that a bottleneck only occurs when both tasks require response selection (Ferreira & Pashler, 2002). While it is difficult to define exactly when response selection occurs during driving, it is conceivable that more responses need to be selected in a difficult driving scenario, in turn leading to greater interference in this condition. Another possibility is that participants were more engaged with the speaking task while driving than when speaking in isolation, possibly making interference effects between speaking-only and easy-driving conditions harder to detect. However, the positive co-variation between task performances remains difficult to explain under these scenarios. If the observed interference was solely due to the need of certain processes to alternate, speaking should either improve when driving declines (negative correlation), or if participants systematically prioritize the same task over the other, the two task performances should be uncorrelated^5^. Under these scenarios, the observed positive co-variation would need to be due to something else. One explanation could be attentional lapses that affect both tasks. Brief episodes where attention shifts away from the task are indeed associated with poorer driving control (Lin et al., 2016). However, mind wandering in driving is more typical for monotonous, low-demand conditions (*highway-hypnosis*, Lin et al., 2016). The results of the Granger causality analysis rather suggest that covariation was more pronounced under difficult driving conditions. Moreover, attention lapses typically accumulate with the time spent on the task (Romer et al., 2014). In our analysis, performance was not significantly impacted by time-on-task, and visualizations do not indicate performance declines over time.

Taken together, the present findings suggest that the interference in speaking observed in a continuous, ecologically valid multitasking setting is best explained by capacity limitations in shared cognitive resources rather than by serial processing. While bottleneck accounts can accommodate parts of the data under specific assumptions, they do not naturally predict the observed covariation between task performances. Importantly, while our findings suggest that the observed interference stems from limited resources, they do not indicate whether serial processing occurred in our setting (without generating interference, or generating interference we could not detect). Moreover, it remains to be examined whether the observed patterns generalize to other ecological tasks.

Besides theoretical advances, the present study introduces a novel way to study performance in ecological, continuous multitasking paradigms. We show that it is feasible and informative to track participants’ performance as the tasks progress. Additional research is now needed to understand how best to track the relationship between continuous task performance on two concurrent tasks, for instance, by defining the optimal length of the time windows used to compute the two time series. Another potential issue of interest concerns the differences in the results of the Granger causality analysis compared to those of linear regression. Whereas in the first, the time series were related only in the hard-driving condition, there was no interaction with task difficulty in the latter. This difference could occur because in the Granger causality analysis, one measure predicts the other in the subsequent time window, whereas in linear regression, they predict one another in the same time window. Alternatively, this difference could stem from the fact that Granger causality captures varying trends throughout the experiment, accounting for both positive and negative relationships depending on the time window, whereas the linear model provides information on the global trend of this relationship over the course of the experiment.

The novel analytical approach introduced here paves the way for new investigations into the interplay between language production and other cognitive subsystems. An important focus of psycholinguistic studies has been to determine *which* of the processes assumed by psycholinguistic models of language production (conceptualization, lexical access, word form encoding, motor planning, e.g., Dell et al., 1997) are (not) functionally independent. This issue is far from settled (Ayora et al., 2011; Cook & Meyer, 2008; Ferreira & Pashler, 2002; Fournet et al., 2021; Paucke et al., 2015) and could be addressed by manipulating the linguistic material in the speaking task, such that different components of language processing can be targeted in ecological settings. Similarly, varying the requirements of the concurrent cognitive task could help determine *which* cognitive abilities are recruited for language production, another debated issue in the field (Fournet et al., 2021; Jongman et al., 2015; Petrone et al., 2011; Shao et al., 2006, 2013).

**Table 5:** Results of Granger causality tests

*Condition*	*p*	*Test*
Easy	0.529	Speech ratio ~ Velocity
Easy	0.87	Velocity ~ Speech ratio
Easy	0.886	Speech ratio ~ Lateral position
Easy	0.847	Lateral position ~ Speech ratio
Hard	<.001	Speech ratio ~ Velocity
Hard	0.001	Velocity ~ Speech ratio
Hard	<.001	Speech ratio ~ Lateral position
Hard	<.001	Lateral position ~ Speech ratio

**Table 6:** Speech ratio as a function of driving condition and variability in lateral position (Latpos) and velocity (Vel)

	Estimate		SE		p	
*Predictor*	*Latpos*	*Vel*	*Latpos*	*Vel*	*Latpos*	*Vel*
Intercept	0.79	0.80	0.01	0.01	<.001	<.001
Condition	0.02	0.01	5.09e-03	5.33e-03	0.004	0.026
Variability	-0.09	-0.08	0.07	0.03	0.214	0.011
Time	2.31e-04	1.62e-04	1.15e-04	1.11e-04	0.053	0.152
Condition*Variability	-0.03	0.04	0.05	0.02	0.625	0.121
Time*Variability	-1.31e-03	-2.16e-04	1.36e-03	6.24e-04	0.351	0.734

## Open practices statement

The anonymized data and analysis scripts for this study are available on the Open Science Framework: https://osf.io/j9ghc/overview?view_only=301c82a4fef24b2db03c2df662db1f8d. None of the experiments was preregistered.

## Figures and Tables

**Fig. 5:**
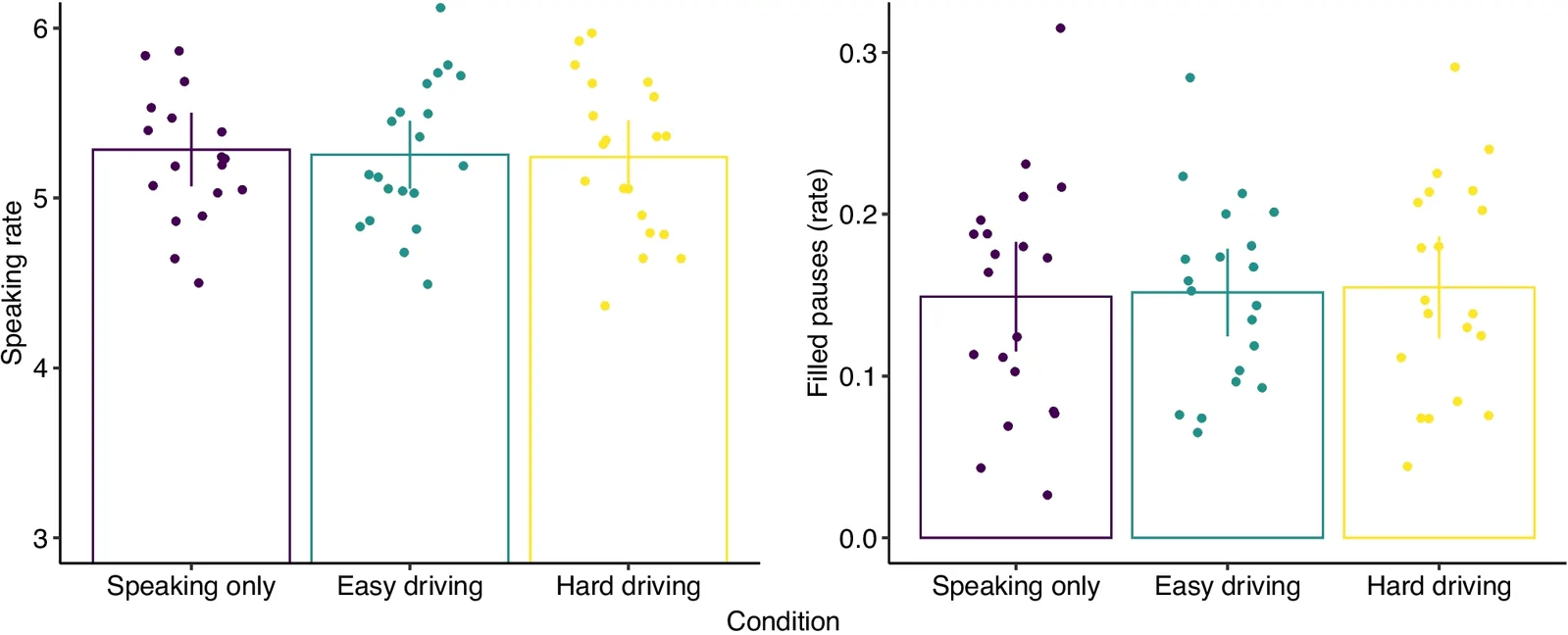
Plots showing speaking rate and the number of filled pauses per condition. Individual *points* represent individual participants. *Error bars* represent 95% confidence intervals

## Data Availability

The materials used for the speaking task are provided in Appendix A. Anonymized participant data are available on the Open Science Framework: https://osf.io/j9ghc/overview?view_only=301c82a4fef24b2db03c2df662db1f8d.

## Appendices

### Appendix A: Prompt lists used for the speaking task

**Table:** German version (original)

Liste A	Liste B	Liste C
Beschreibe einen für dich typischen Dienstag.	Beschreibe einen für dich typischen Sonntag.	Beschreibe deine Morgenroutine unter der Woche und am Wochenende.
Beschreibe deine Wohnung (oder dein Haus) und wer dort mit dir lebt.	In was für einem Gebäude wohnst du und wer wohnt dort noch?	Beschreibe dein Schlafzimmer. Wie ist es eingerichtet?
Was ist (eine) deine(r) liebsten Landesküchen und was magst du daran besonders?	Was für Musik hörst du gerne und warum bevorzugst du diese Musikrichtung anderen gegenüber?	Wer ist dein/e Lieblingsmusiker:in und warum?
Bevorzugst du das Leben auf dem Land oder in der Stadt und warum?	Liest du lieber Bücher oder siehst du lieber Filme und warum?	Bevorzugst du das Meer oder die Berge und warum?
Bist du in deinem Leben schon öfter umgezogen und welche Wohnsituation fandest du bisher am besten?	Hast du bisher schon viele unterschiedliche Jobs gehabt und welchem mochtest du am liebsten?	Was war dein Lieblingsschulfach und warum?
Woran merkst du wenn es Frühling wird, generell sowie speziell in der Region, in der du lebst?	Wie würdest du die Auswirkungen der beginnenden Sommerferien in Deutschland und in der Region, in der du lebst, beschreiben?	Was ist deine Lieblingsjahreszeit? Und warum?
Beschreibe deine Frühstücksroutine an einem Wochentag und am Wochenende.	Welche Kleidung trägst du bevorzugt im Sommer und welche im Winter?	Beschreibe deine Routine zum ins Bett gehen unter der Woche und am Wochenende.
Bist du Kaffee- oder Tee-Trinker:in und warum?	Wie unterscheiden sich Hunde und Katzen und bevorzugst du eine der beiden Tierarten?	Bist du eher ein/e Früh- oder Spätaufsteher:in und würdest du das ändern, wenn du es ändern könntest?
Welche Orte besuchst du am häufigsten und wie gelangst du dorthin?	Gibt es ein bestimmtes Gericht, das du besonders oft oder besonders gerne isst? Bereitest du es selbst zu oder wo isst du es normalerweise?	Gibt es ein bestimmtes Dessert, das du besonders oft oder besonders gerne isst? Bereitest du es selbst zu oder wo isst du es normalerweise?
Wie sieht ein perfekter Geburtstag für dich aus?	Wie sieht ein perfekter Urlaub für dich aus?	Was ist eine Reise, die du gerne machen würdest?
Beschreibe eine Erinnerung aus deiner Kindergartenzeit und warum sie dir im Gedächtnis geblieben ist.	Was ist eine Lieblingsreise, die du gemacht hast?	Beschreibe eine Erinnerung aus deiner Schulzeit und warum sie dir im Gedächtnis geblieben ist.
Fährst du lieber Auto oder nutzt du lieber die öffentlichen Verkehrsmittel?	Reist du lieber mit dem Zug oder mit dem Flugzeug?	Was ist deine Lieblingstageszeit und warum?
Beschreibe deine Küche. Wie ist sie eingerichtet?	Was unternimmst du gerne mit deinen Freunden?	Was unternimmst du gerne mit deiner Familie?
Wenn du ein beliebiges Tier sein könntest, welches wärst du gerne?	Wenn du eine beliebige fiktive Person sein könntest, wer wärst du gerne?	Könntest du dir vorstellen, Veganer:in zu sein?
Könntest du dir vorstellen, Vegetarier zu sein?	Welche Stadt würdest du gerne besuchen?	Welches Land würdest du gerne bereisen?
Wie entspannst du dich nach einem langen Arbeitstag?	Wie entspannst du dich an einem freien Tag?	Was ist deine Lieblingssportart?

**Table:** English version (translation)

List A	List B	List C
Describe a typical Tuesday for you.	Describe a typical Sunday for you.	Describe your morning routine on weekdays and weekends.
Describe your apartment (or house) and who lives there with you.	What kind of building do you live in, and who else lives there?	Describe your bedroom. How is it furnished?
What is one of your favorite national cuisines, and what do you particularly like about it?	What kind of music do you enjoy, and why do you prefer this genre over others?	Who is your favorite musician, and why?
Do you prefer living in the countryside or in the city, and why?	Do you prefer reading books or watching movies, and why?	Do you prefer the sea or the mountains, and why?
Have you moved frequently in your life, and which living situation did you like best so far?	Have you had many different jobs, and which one did you like the most?	What was your favorite school subject, and why?
How do you notice when spring begins, both generally and in your specific region?	How would you describe the effects of the beginning of summer vacation in Germany and in your region?	What is your favorite season, and why?
Describe your breakfast routine on a weekday and on the weekend.	What clothes do you prefer to wear in summer and in winter?	Describe your bedtime routine on weekdays and weekends.
Are you a coffee or tea drinker, and why?	How do dogs and cats differ, and do you prefer one over the other?	Are you an early bird or a night owl, and would you change that if you could?
What places do you visit most often, and how do you get there?	Is there a particular dish you eat frequently or enjoy the most? Do you prepare it yourself, or where do you usually eat it?	Is there a specific dessert you eat often or enjoy the most? Do you prepare it yourself, or where do you usually eat it?
What does a perfect birthday look like for you?	What does a perfect vacation look like for you?	What is a trip you would like to take?
Describe a memory from your kindergarten years and why it has stayed in your mind.	What is a favorite trip you have taken?	Describe a memory from your school years and why it has stayed in your mind.
Do you prefer driving a car or using public transportation?	Do you prefer traveling by train or by plane?	What is your favorite time of day, and why?
Describe your kitchen. How is it furnished?	What do you enjoy doing with your friends?	What do you enjoy doing with your family?
If you could be any animal, which one would you like to be?	If you could be any fictional character, who would you like to be?	Could you imagine being vegan?
Could you imagine being vegetarian?	Which city would you like to visit?	Which country would you like to travel to?
How do you relax after a long workday?	How do you relax on a day off?	What is your favorite sport?

### Appendix B: Results of exploratory analyses of speaking rate and the number of filled pauses

Exploratory analyses were run on two additional speaking measures, speaking rate and the number of filled pauses. A mixed-effects Poisson regression model revealed no significant differences in speaking rate between speaking-only and the driving conditions, or the easy and hard driving condition (see Table 7 and Fig. 5).

We ran another mixed-effects Poisson regression to predict the rate of filled pauses relative to the time spent speaking. The number of filled pauses produced did not differ significantly between the speaking-only and the driving conditions, or between easy and hard driving (see Table 8 and Fig. 5). The average response time on the PDT task predicted the number of filled pauses, indicating that participants produced fewer filled pauses when the driving task was more automatic for them.

**Table 7:** Differences in speaking rate between conditions

*Predictors*	*Estimate*	*SE*	*p*
Intercept	1.59	0.13	<.001
Speaking only - Driving	0.01	7.60e-03	0.106
Easy driving - Hard driving	2.98e-03	8.64e-03	0.73
Response time PDT	0.16	0.28	0.573

**Table 8:** Differences in number of filled pauses between conditions

*Predictors*	*Estimate*	*SE*	*p*
Intercept	-1.97	0.09	<.001
Speaking only - Driving	-0.02	0.04	0.688
Easy driving - Hard driving	-0.03	0.05	0.589
Response time PDT	2.78	1.44	0.053

### Appendix C: Results of the linear mixed models predicting variability in velocity and lateral position as a function of speech ratio

We used two linear mixed models to predict variability in velocity and variability in the lateral position of the car on the road as a function of driving condition, speech ratio, their interaction, and a predictor controlling for the effect of time on task. The factor for condition was coded with a scaled sum contrast. Speech ratio was centered on the mean. The models were run with correlated by-participant varying intercepts and slopes. Model outputs can be found in Tables 9 and 10. For both lateral position and velocity, variability was lower in the easy than in the hard driving condition, and there was a negative relationship with speech ratio. Whenever driving becomes less variable (i.e., more efficient), speaking becomes more fluent (i.e., speech ratio becomes higher).

We replicate the reciprocal interference (from speaking while driving and from driving while speaking; see the main analysis that modeled speech ratio as a function of driving variability) observed in previous studies, further supporting the notion that participants do not systematically prioritize the same task throughout the experiment.

**Table 9:** Variability in lateral position as a function of driving condition and speech ratio

*Predictors*	*Estimate*	*SE*	*p*
Intercept	0.17	9.17e-03	<.001
Condition	-0.02	6.70e-03	0.007
Speech ratio	-0.06	0.05	0.245
Time	-1.04e-04	1.04e-04	0.326
Condition*Speech ratio	5.21e-03	0.03	0.883
Time*Speech ratio	-8.15e-04	1.01e-03	0.427

**Table 10:** Variability in velocity as a function of driving condition and speech ratio

*Predictors*	*Estimate*	*SE*	*p*
Intercept	0.29	0.03	<.001
Condition	-0.09	0.02	0.001
Speech ratio	-0.29	0.14	0.061
Time	-8.03e-04	4.22e-04	0.073
Condition*Speech ratio	0.32	0.13	0.029
Time*Speech ratio	-2.42e-03	3.35e-03	0.481

## Footnotes

1 Other studies in which task difficulty is manipulated in similar settings target the encoding and retrieval of information in memory (Becic et al., 2010), the comprehension of relative clauses (Engonopulos & Demberg, 2013), or spatial reasoning (Kubose et al., 2006) rather than language planning.

2 For a model that predicted variability in driving measures by speech ratio, see Appendix C.

3 The predictor *time* was included to control for potential practice and fatigue effects. Because the hard-driving condition was always performed last, participants had more practice with the task by the time they performed this condition. Despite this, performance was systematically poorer in the hard than in the easy driving condition.

4 In addition to the interference from driving on speaking, we also found interference from speaking on driving in an exploratory analysis reported in Appendix C. These findings refute the hypothesis that a single task was prioritized over the other throughout the experiment.

5 A reviewer suggested that general performance collapses may occur on top of serial processing, leading to simultaneous declines in both speaking and driving, thereby producing a positive relationship between the two. We note that this explanation effectively assumes that task demands occasionally exceed available cognitive resources – equivalent to the assumptions of capacity-limited accounts.

## Code Availability

The analysis scripts used for this study are available on the Open Science Framework: https://osf.io/j9ghc/overview?view_only=301c82a4fef24b2db03c2df662db1f8d.
